# Phase 2 study of canfosfamide in combination with pegylated liposomal doxorubicin in platinum and paclitaxel refractory or resistant epithelial ovarian cancer

**DOI:** 10.1186/1756-8722-3-9

**Published:** 2010-03-11

**Authors:** John J Kavanagh, Charles F Levenback, Pedro T Ramirez, Judith L Wolf, Carla L Moore, Marsha R Jones, Lisa Meng, Gail L Brown, Robert C Bast

**Affiliations:** 1 The MD Anderson Cancer Center, University of Texas, Houston, TX, USA; 2 Telik, Inc, Palo Alto, CA USA

## Abstract

**Background:**

Canfosfamide is a novel glutathione analog activated by glutathione S-transferase P1-1. This study evaluated the safety and efficacy of canfosfamide in combination with pegylated liposomal doxorubicin (PLD) in patients with platinum resistant ovarian cancer. Patients with platinum resistant ovarian carcinoma and measurable disease received canfosfamide at 960 mg/m^2 ^in combination with PLD at 50 mg/m^2^, intravenously day 1 in every 28 day cycles until tumor progression or unacceptable toxicities. The primary endpoints were objective response rate (ORR) and progression-free survival (PFS).

**Results:**

Canfosfamide plus PLD combination therapy was administered at 960/50 mg/m^2^, respectively. Thirty-nine patients received a median number of 4 cycles (range 1.0-18.0). The ORR was 27.8% (95% CI, 14.2-45.2) with a disease stabilization rate of 80.6% (95% CI, 64.0-91.8) in the evaluable population. The CA-125 marker responses correlated with the radiological findings of complete response or partial response. The median PFS was 6.0 months (95% CI, 4.2-7.9) and median survival was 17.8 months. The combination was well tolerated. Myelosuppression was managed with dose reductions and growth factor support. Grade 3 febrile neutropenia was observed in 2 patients (5.1%). Non-hematologic adverse events occurred at the expected frequency and grade for each drug alone, with no unexpected or cumulative toxicities.

**Conclusions:**

Canfosfamide in combination with PLD is well tolerated and active in platinum and paclitaxel refractory or resistant ovarian cancer. A randomized phase 3 study was conducted based on this supportive phase 2 study.

**Trial Registration:**

This study was registered at www.clinicaltrials.gov: NCT00052065.

## Background

Ovarian cancer accounts for approximately 3% of all cancers in women and is the fifth leading cause of cancer-related deaths among women with an estimated 22,000 new cases and 14,600 deaths in the U.S. in 2009 [1]. The standard initial treatment of patients with advanced ovarian cancer, cytoreductive surgery, followed by combination chemotherapy with platinum and paclitaxel, has resulted in response rates of 70% and a median survival of 37 months [2,3]. Despite the activity of this combination chemotherapy as first-line treatment, the majority of patients experience recurrence and die of chemotherapy-resistant disease [4]. One of the challenges confronting oncologists is the management of persistent or recurrent platinum resistant disease.

Platinum refractory or resistant ovarian cancer is defined by the Gynecologic Oncology Group (GOG) as persistent disease or progression within 6 months following platinum-based therapy, and is associated with a low response rate to further treatment, responses of short duration, and a median survival of less than 1 year. The treatment options for platinum resistant patients are limited [5]. The most widely used approved drugs for this indication are topotecan and pegylated liposomal doxorubicin (PLD). A randomized phase 3 trial comparing these agents showed modest improvement in survival for PLD as compared to topotecan in platinum-sensitive patients [6]. However, in the platinum resistant population, the objective response rates (ORR) for PLD and topotecan were 12.3% and 6.5%, respectively, which correlated with a median progression-free survival (PFS) of 9.1 weeks and 13.6 weeks and a median survival of 35.6 weeks and 41.3 weeks, respectively [7]. The frequencies of grade 4 drug related adverse events (AEs) were 71.1% for topotecan and 17.2% for PLD. Combination chemotherapy has not been demonstrated to be better than single-agent therapy in the few small phase 2 studies performed in platinum resistant ovarian cancer. These studies reported increased toxicity without an impact on survival in this population. Platinum resistant ovarian cancer continues to represent a significant unmet medical need requiring the development of new agents and regimens.

Canfosfamide HCl for injection (TELCYTA^®^, TLK286), a novel glutathione analog, is currently being developed for the treatment of cancer. Canfosfamide is a conjugate of a glutathione (GSH) analog and an N,N,N',N'-tetrakis(2-chloroethyl) phosphorodiamidate that was designed to be metabolically activated by glutathione-S-transferase P1-1 (GST P1-1), an enzyme that is over-expressed in many human cancers including ovarian cancer. The active cytotoxic phosphorodiamidate is released after cleavage by GST P1-1 [8-13]. Canfosfamide treatment, therefore, may result in selective delivery of the cytotoxic moiety to ovarian cancer cells by exploiting the elevated enzymatic activity of GST P1-1 present in these cells.

Preclinical studies showed that canfosfamide inhibited the growth and was cytotoxic to a wide range of established cancer cell lines including those derived from ovarian cancer (OVCAR3, HEY, SK-OV-3) [14,15]. Canfosfamide treatment inhibited cancer cell proliferation and induced apoptosis through the activation of the cellular stress response kinase pathway. The molecular events that preceded apoptosis included the activation of stress-activated kinases, including the phosphorylation of the mitogen-activated protein kinase (MAPK) signaling protein, mitogen activated protein kinase kinase 4 (MKK4), in canfosfamide treated cells, as well as the activation of jun-N-terminal kinase (JNK), p38 MAP kinase and caspase 3 [14,16].

The cytotoxic activity of canfosfamide correlated with the expression of GST P1-1. Cancer cells in which GST expression levels were increased by transfection with the GST P1-1 gene, were more sensitive to the cytotoxic effects of canfosfamide than the parental cell lines [16,17]. Canfosfamide exhibited increased cytotoxic activity in vitro and in vivo against tumors derived from cancer cells and induced to express elevated levels of GST P1-1, including those with elevated GST P1-1 as a result of acquired resistance to doxorubicin [16]. Canfosfamide treatment inhibited tumor growth in a range of established human cancer xenografts including those derived from human ovarian cancer.

Canfosfamide was not cross-resistant to carboplatin, cisplatin or paclitaxel in OVCAR3 human ovarian cancer cells [18]. Canfosfamide treatment synergistically enhanced the cytotoxicity in vitro of a variety of chemotherapeutic agents with different modes of action, including carboplatin, doxorubicin, paclitaxel, and gemcitabine [19].

In addition to its favorable preclinical profile, canfosfamide has additional attributes that suggest it would be of interest to evaluate its clinical activity in combination with PLD in platinum resistant ovarian cancer. Canfosfamide has shown single-agent activity in heavily pretreated platinum resistant ovarian cancer patients with an ORR of 15% (95% CI, 5-31) and 19% (95% CI, 7-36) on 2 dose schedules of every 3 weeks and weekly therapy, respectively, by Response Evaluation Criteria In Solid Tumors (RECIST), including a durable complete response (CR) in a platinum refractory patient [20,21]. Canfosfamide has been well tolerated in these patients, who often have limited bone marrow reserves or neuropathic residual toxicities. Canfosfamide is generally non-myelosuppressive at the recommended dose and dose schedule and does not have overlapping toxicities with PLD suggesting that canfosfamide should not compromise the dose of PLD. In addition to the preclinical synergy observed with the combination of canfosfamide and doxorubicin in human ovarian cancer cells [19], doxorubicin has been shown in vitro to increase the expression of GST P1-1 [22], and consequently, facilitates the activation of canfosfamide. Since canfosfamide has shown activity over a wide range of dose schedules, a clinically-convenient dose schedule of canfosfamide in combination with PLD could be administered every 4 weeks. A phase 1 dose escalation study of canfosfamide at a dose of 500 mg/m^2^, 750 mg/m^2^, or 960 mg/m^2 ^was administered intravenously (IV) followed by PLD at 40 or 50 mg/m^2 ^IV every four weeks. The primary endpoints of the Phase 1 study were to determine the safety and maximum tolerated dose (MTD) of the combination. There were no dose limiting toxicities (DLTs). The MTD was full doses of both agents. Full doses of canfosfamide in combination with PLD were administered in 88.4% and 87.3% of cycles, respectively. The most common reasons for dose reductions were neutropenia and/or thrombocytopenia as expected with PLD administered at 50 mg/m^2^. There were no unexpected or cumulative toxicities [23].

Based on the above rationale, this study was conducted to evaluate the safety and efficacy of the combination of canfosfamide with PLD in patients with platinum resistant ovarian cancer.

## Materials and methods

All patients provided written informed consent prior to their participation in the study. In this single institution study, the protocol was approved by the M.D. Anderson Cancer Center, University of Texas institutional review board and reviewed annually. The study was conducted in accordance with the International Conference on Harmonization Good Clinical Practice standards. In this phase 2 study, the Sponsor provided a safety monitoring plan for the study and the Sponsor's safety, pharmacovigilance committee was responsible for the oversight of the safety of the study participants. The Principal Investigator was responsible for selection of candidate patients.

### Patients

Women who were at least 18 years old with recurrent, histologically confirmed epithelial ovarian, primary peritoneal, or fallopian tube cancer; measurable disease as defined by RECIST; had received at least 1 but fewer than 4 prior platinum-containing chemotherapy regimens; at least 1 prior paclitaxel-containing regimen; and considered platinum refractory or resistant disease according to the standard GOG criteria (had progressed during or had persistent disease after completion of platinum-based therapy or had a platinum-free interval of < 6 months) were enrolled. There were no additional limits to lines of therapy. Other requirements included an Eastern Cooperative Oncology Group (ECOG) performance status of 0 to 2, adequate bone marrow reserve defined as an absolute neutrophil count (ANC) ≥ 1500/mm^3^, platelet count ≥ 100,000/mm^3^, and hemoglobin ≥ 9.0 g/dL, total bilirubin < 1.2 mg/dL, creatinine < 1.5 mg/dL or a calculated creatinine clearance of at least 60 mL/min, alanine amino-transferase < 3 times upper limit of normal and adequate cardiac function [left ventricular ejection fraction (LVEF) of ≥ 50% of the institutional normal and New York Heart Association classification Class I or II] or signs of intestinal obstruction interfering with nutrition.

### Procedures

Canfosfamide was administered as a 30-minute constant rate intravenous (IV) infusion on day 1 of each 4-week cycle at 960 mg/m^2 ^followed by PLD at 50 mg/m^2 ^IV at an initial rate of infusion of 1 mg/min. If no acute infusion reactions occurred, subsequent doses of PLD were administered over 1 hour. Treatment cycles were repeated every 4 weeks until tumor progression. Cycles of therapy could be postponed up to 4 weeks due to toxicity; longer toxicity delays led to study treatment discontinuation. Premedications and the use of growth factor and transfusion support were permitted.

Patients were assessed at baseline and every cycle during treatment. The baseline assessments included: medical history, physical examination, ECOG performance status, complete blood count with differential and platelet count, chemistry profile, electrocardiogram (ECG), spiral/helical computed tomography (CT) or magnetic resonance imaging (MRI) scans of all areas of metastatic disease to establish the extent of tumor burden with documentation of tumor measurements by RECIST, CA-125 tumor marker, urinalysis, and pregnancy test. Toxicity was assessed every cycle and until 30 days after the last study treatment; nadir blood counts were obtained between days 8 and 15 of every cycle. During treatment, medical history, physical examination, and chemistries including creatinine, total bilirubin, electrolytes, alkaline phosphatase, serum glutamic oxaloacetic transaminase, serum glutamate pyruvate transaminase, albumin and CA-125 were performed every 4 weeks. Tumor assessments by RECIST were obtained every 2 cycles or 8 weeks. The responses were confirmed with independent radiology review (IRR) as well as being read centrally at the site. All sites of metastatic disease were assessed using the same methods as those used at baseline. Objective tumor responses (CRs or PRs) were confirmed by CT or MRI scans within 4 to 6 weeks after the first documented response. All patients with PR or stable disease (SD) continued to receive treatment and underwent CT or MRI scans every 2 cycles or 8 weeks until evidence of tumor progression or unacceptable toxicities occurred. At the investigator's discretion, patients with CR received a minimum of 2 additional cycles beyond documentation of CR. Adverse events (AEs) were graded using the National Cancer Institute-Common Toxicity Criteria Version 2.0 (NCI-CTC v2.0) [24].

### Dose Adjustments

Dose adjustments for canfosfamide were required for the following toxicities: ≥ grade 3 hematologic toxicity; ≥ grade 3 toxicity impacting organ function other than alopecia, nausea, and vomiting. Dose modifications for PLD were based upon the PLD prescribing information: ≥ grade 3 hematologic toxicity; ≥ grade 2 palmar-plantar erythrodysesthesia (PPE); ≥ grade 2 stomatitis; or changes in liver function as measured by serum bilirubin. Treatment resumed after recovery from non-hematologic and hematologic toxicities (ANC ≥ 1.5 × 10^9^/L and platelets ≥ 100 × 10^9^/L).

### Statistical Methods

All treated patients were considered as intent-to-treat (ITT) and evaluated in the safety and efficacy analyses. All patients who received any amount of study drug(s) were included in the safety population for AE analysis, which was graded according to NCI-CTC v2.0. A patient must have had adequate baseline tumor assessment, received 2 cycles of study treatment and had at least 1 follow-up tumor assessment for response to be included in the efficacy evaluable (EE) population.

Patient demographics and ovarian cancer disease characteristics and AEs were evaluated using descriptive statistics in terms of count and percentage for categorical variables and sample size, mean, median, and range for continuous variables. Event variables were calculated as rates with the exact binomial 95% confidence intervals provided. Progression-free survival was defined as from the date of cycle 1 day 1 study treatment dosing until the date of tumor progression or death from any cause, whichever occurred first. Overall survival was determined from the date of cycle 1 day 1 study treatment dosing to the date of death from any cause. Progression-free survival and overall survival were summarized using the Kaplan-Meier method [25].

## Results

### Patient Demographics and Ovarian Cancer Disease Characteristics

From January 27, 2003, to July 20, 2004, 39 patients received canfosfamide at 960 mg/m^2 ^and PLD at 50 mg/m^2 ^every 4 weeks. Patient demographics, baseline characteristics, and prior therapies are shown in Table 1. Patients had a median age of 54.5 years (range 34.8 to 75.4) and 32 patients (82.1%) had an ECOG performance status of 0.

**Table 1:** Patient demographics and ovarian cancer disease characteristics (N = 39)

**Age**		**Baseline Platinum Status**	**n (%)**
Median	54.5	Platinum Refractory or Primary Resistant	15 (38.5)
Range	34.8-75.4	Secondary Platinum Resistant	24 (61.5)
**Cancer Diagnosis**	**n (%)**	**Bulky Disease**	
Ovary	37 (94.9)	Present	11 (28.2)
Peritoneal	1 (2.6)	Absent	27 (69.2)
Fallopian Tube	1 (2.6)	Unknown	1 (2.6)
**ECOG Performance Status**		**Ascites**	
0	32 (82.1)	Present	7 (17.9)
1	5 (12.8)	Absent	32 (82.1)
2	1 (2.6)	**Number of prior therapies**	
NA	1 (2.6)	Median (range)	4.0 (2.0-10.0)
**FIGO Stage at Initial Diagnosis**		**Number of prior cancer surgery**	**n (%)**
IA	1 (2.6)	1	25 (64.1)
IC	1 (2.6)	2	13 (33.3)
IIC	3 (7.7)	3	1 (2.6)
III	3 (7.7)	**Number of prior radiation therapy**	
IIIA	1 (2.6)	0	35 (89.7)
IIIB	2 (5.1)	1	3 (7.7)
IIIC	15 (38.5)	2	1 (2.6)
IV	4 (10.3)	**Number of platinum-containing regimens**	
Unknown	9 (23.1)	1	14 (35.9)
**Race/Ethnicity**		2	17 (43.6)
Caucasian	34 (87.2)	3	5 (12.8)
Black	2 (5.1)	4	2 (5.1)
Asian	2 (5.1)	5	1 (2.6)
Hispanic	1 (2.6)	**Number of prior chemotherapy regimens (counting all prior platinum-containing regimens as one)**	
Other	0 (0.0)		
		1	16 (41.0)
Histologic Subtype*		2	11 (28.2)
Serous papillary	30 (76.9)	3	4 (10.3)
Mucinous	0 (0.0)	4	3 (7.7)
Poorly Differentiated	2 (5.1)	5	4 (10.3)
Endometrioid	3 (7.7)	6	1 (2.6)
Clear Cell	7 (17.9)	Prior Chemotherapy*	
Mixed	4 (10.3)	Platinum and Paclitaxel	39 (100.0)
**Baseline CA125 (U/mL)**		Topotecan	9 (23.1)
Median	178.4	Docetaxel	12 (30.8)
Range	7.7--9321.1	Gemcitabine	11 (28.2)

*Not mutually exclusive

The primary tumor site was ovary in 37 of 39 patients (94.9%) and the most common histology was serous papillary (76.9%). The median CA-125 level at baseline was 178.4 (range 7.7-9321.1). Eleven patients (28.2%) had known bulky disease defined as having at least 1 tumor ≥ 5 cm present and 7 patients (17.9%) had ascites. The best response to prior platinum-based therapy was CR in 61.5%, PR in 20.5%, SD in 5.1%, and progressive disease (PD) in 12.8%.

These patients had been heavily treated with a median number of 4 prior therapies (range 2-10). Fifteen patients (38.5%) were platinum refractory or primary resistant and 24 patients (61.5%) had secondary platinum resistant disease. All patients (100%) were platinum and paclitaxel refractory or resistant. All patients had received additional non-platinum containing salvage agents, including docetaxel in 12 (30.8%), gemcitabine in 11 (28.2%) and topotecan in 9 (23.1%). All prior platinum-containing regimens were counted as 1 regimen. Most patients (59.0%) received 2 or more prior chemotherapy regimens (median 2; range 1-6). Twelve patients (30.8%) received 3 or more and 8 patients (20.5%) received 4 or more prior regimens, defining a heavily-treated population.

### Study Treatment Administration

Thirty-nine patients received a total of 245 cycles of canfosfamide in combination with PLD therapy as shown in Table 2. The median number of cycles per patient was 4 (range 1-18). The median cumulative dose of canfosfamide was 3840 mg/m^2 ^(range 960-13,978 mg/m^2^) and of PLD 200.3 mg/m^2 ^(range 50.0-726.4 mg/m^2^). Full doses of canfosfamide and PLD were administered in 88.4% and 87.3% of cycles, respectively. Dose reductions due to toxicity were infrequent. The most common reasons for dose reductions were 14 events of PPE syndrome and 28 events of neutropenia and/or thrombocytopenia.

**Table 2:** Canfosfamide and pegylated liposomal doxorubicin treatment administration and adjunctive care (N = 39)

**Dosing (Total # of Cycles = 245)**	**Canfosfamide**	**PLD**
Median # Cycles/Patient (range)	4.0 (1-18.0)	
Median Cumulative Dose, mg/m^2 ^(range)	3840 (960.0-13978)	200.3 (50.0-726.4)
Dose Reductions	28	31
Dose Interruptions	14	6
**Adjunctive Treatment**	**# Cycles**	**% Cycles**
Granulocyte Growth Factor Support	79	32.2
Erythropoietin Support	49	20.0
RBC Transfusions	18	7.3
Platelet Transfusions	1	0.4

### Safety

Treatment-related AEs related to the combination of canfosfamide and PLD are shown in Table 3. Grade 4 hematologic AEs included neutropenia [11 patients (28.2%)], leucopenia [2 patients (5.1%)], and anemia [1 patient (2.6%)]. Febrile neutropenia (grade 3) was observed in 2 patients (5.1%). Granulocyte growth factor was administered in 32.2% of cycles and erythropoietin was administered in 20% of cycles. Red blood cell transfusions were given in 7.3% of cycles and a single platelet transfusion was administered in 0.4% of cycles. Two patients with neutropenic fever received granulocyte colony stimulating factor (G-CSF) for 3 and 7 days, respectively, with prompt resolution of neutropenia. There were no reports of treatment-related sepsis or clinical sequelae.

**Table 3:** Adverse events related to the canfosfamide and pegylated liposomal doxorubicin combination (NCI-CTC v2.0) (N = 39)

	Grade 1 n (%)	Grade 2 n (%)	Grade 3 n (%)	Grade 4 n (%)
**Hematologic (All Patients)**				
Anemia	9 (23.1)	21 (53.8)	5 (12.8)	1 (2.6)
Leucopenia	3 (7.7)	13 (33.3)	15 (38.5)	2 (5.1)
Neutropenia	0 (0.0)	8 (20.5)	12 (30.8)	11 (28.2)
Thrombocytopenia	10 (25.6)	5 (12.8)	10 (25.6)	0 (0.0)
Leukocytosis	8 (20.5)	0 (0.0)	0 (0.0)	0 (0.0)
Febrile Neutropenia	0 (0.0)	0 (0.0)	2 (5.1)	0 (0.0)
**Non-hematologic (≥ 5% of patients)**				
Nausea	9 (23.1)	20 (51.3)	6 (15.4)	0 (0.0)
Fatigue	1 (2.6)	11 (28.2)	20 (51.3)	1 (2.6)
Vomiting	10 (25.6)	8 (20.5)	4 (10.3)	0 (0.0)
Rash	8 (20.5)	12 (30.8)	3 (7.7)	0 (0.0)
Diarrhea	1 (2.6)	6 (15.4)	3 (7.7)	0 (0.0)
Drug Hypersensitivity	1 (2.6)	1 (2.6)	1 (2.6)	0 (0.0)
Infusion Site Pain	4 (10.3)	0 (0.0)	0 (0.0)	0 (0.0)
Pyrexia	2 (5.1)	2 (5.1)	0 (0.0)	0 (0.0)
Dysuria**	1 (2.6)	1 (2.6)	0 (0.0)	0 (0.0)
Stomatitis*	4 (10.3)	16 (41.0)	1 (2.6)	0 (0.0)
PPE Syndrome*	3 (7.7)	10 (25.6)	6 (15.4)	0 (0.0)
Mucosal Inflammation*	8 (20.5)	11 (28.2)	0 (0.0)	0 (0.0)
Alopecia*	16 (41.0)	1 (2.6)	0 (0.0)	0 (0.0)
Neuropathy*	3 (7.7)	2 (5.1)	2 (5.1)	0 (0.0)
Pain in Extremity*	2 (5.1)	3 (7.7)	3 (7.7)	0 (0.0)
Erythema*	5 (12.8)	2 (5.1)	0 (0.0)	0 (0.0)
Dry Skin*	2 (5.1)	2 (5.1)	0 (0.0)	0 (0.0)
Pruritus*	3 (7.7)	0 (0.0)	0 (0.0)	0 (0.0)
Gingivitis*	1 (2.6)	1 (2.6)	0 (0.0)	0 (0.0)
Dermatitis*	0 (0.0)	1 (2.6)	1 (2.6)	0 (0.0)
Pigmentation Disorder*	1 (2.6)	1 (2.6)	0 (0.0)	0 (0.0)
Flushing*	2 (5.1)	0 (0.0)	0 (0.0)	0 (0.0)

*Related to pegylated liposomal doxorubicin only

**Related to canfosfamide only

The most common non-hematological AEs related to the combination of canfosfamide and PLD were grade 1-2 and included: nausea (74.4%) and vomiting (46.1%) which were well controlled with standard prophylactic antiemetics, rash (51.2%), and grade 3 fatigue (51.3%). One patient (2.6%) experienced grade 4 fatigue. There were no signs or symptoms of congestive heart failure and no changes in LVEF as determined by multiple gated acquisition or ECG. No treatment-related deaths occurred. The non-hematologic AEs occurred at the expected frequency and grade for each drug alone, with no unexpected or cumulative toxicities.

### Efficacy

Thirty-nine patients were in the ITT population. Thirty-six patients received at least 2 cycles of canfosfamide with PLD combination therapy, had an adequate baseline tumor assessment, and at least 1 follow-up scan, defining the EE population.

An ORR by RECIST of 25.6% (95% CI, 13.0-42.1) in the ITT population and 27.8% (95% CI, 14.2-45.2) in the EE population was reported (Table 4). One CR (2.8%) and 9 partial responses (PRs) (25%) were reported. Patients with platinum refractory and primary resistant disease had comparable ORR to patients with secondary platinum resistant disease. Patients who were assessed as CR or PR had decrements in CA-125 tumor markers commensurate with their tumor responses. The median time to objective response was 2.9 months and the median duration of response was 9.7 months.

**Table 4:** Efficacy

Population	Intent-to-Treat (N = 39)		Efficacy Evaluable (N = 36)	
	n (%)	95% CI	n (%)	95% CI
**Objective Response**	10 (25.6)	13.0-42.1	10 (27.8)	14.2-45.2
CR	1 (2.6)	0.1-13.5	1 (2.8)	0.1-14.5
PR	9 (23.1)	11.1-39.3	9 (25.0)	12.1-42.2
SD	20 (51.3)	34.8-67.6	19 (52.8)	35.5-69.6
PD	8 (20.5)	9.3-36.5	7 (19.4)	8.2-36.0
NE*	1 (2.6)	----	0	0
DSR	30 (76.9)	60.7-88.9	29 (80.6)	64.0-91.8
Patients having SD ≥ 3 months	19 (48.7)		19 (52.8)	
**Objective Response by RECIST by Platinum Status (ITT)**	**Platinum Refractory or Primary Platinum Resistant** **N = 15 n (%); [95% CI]**		**Secondary Platinum Resistant** **N = 24 n (%); [95% CI]**	
ORR	4 (26.7); [7.8-55.1]		6 (25.0); [9.8-46.7]	
CR	1 (6.7); [0.2-31.9]		0	
PR	3 (20.0); [4.3-48.1]		6 (25.0); [9.8-46.7]	
SD	8 (53.3); [26.6-78.7]		12 (50.0); [29.1-70.9]	
PD	3 (20.0); [4.3-48.1]		5 (20.8); [7.1-42.2]	
NE	0		1 (4.2); NA	
Patients having SD ≥ 3 Months	7 (46.7)		12 (50.0)	
	**ITT Population** **n; Median**		**ITT Population** **Q1-Q3**	
**Duration of Response (Months)**	10; 9.7		5.8-NA	
CR	1; NA		NA	
PR	9; 9.7		5.8-NA	
**Duration of SD**	20; 6.4		4.3-13.9	
**Time to Objective Response**	10; 2.9		2.3-3.9	
	**Platinum Refractory or Primary Platinum Resistant** **N = 15**	**Secondary Platinum Resistant** **N = 24**	**All** **N = 39**	
	Median (Q1-Q3)	Median (Q1-Q3)	Median (Q1-Q3)	
**Time to Tumor Progression (Months)**	6.4 (4.1-14.0)	6.0 (2.6-12.0)	6.2 (3.3-12.0)	
**Progression-free Survival (Months)**	6.4 (4.1-14.0)	5.8 (2.3-11.6)	6.0 (2.6-12.0)	
**Overall Survival (Months)**	17.8 (7.0-NA)	17.4 (8.1-NA)	17.8 (7.7-NA)	

Abbreviations: NE, not evaluable; CI, confidence interval

**Figure 1:**
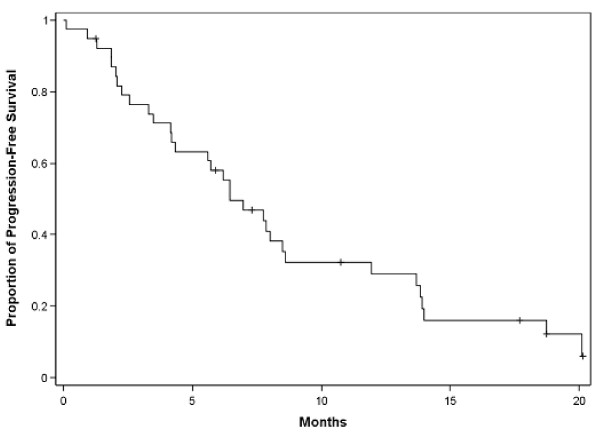
**Progression-free survival (PFS) in patents with platinum refractory or resistant epithelial ovarian cancer**.

**Figure 2:**
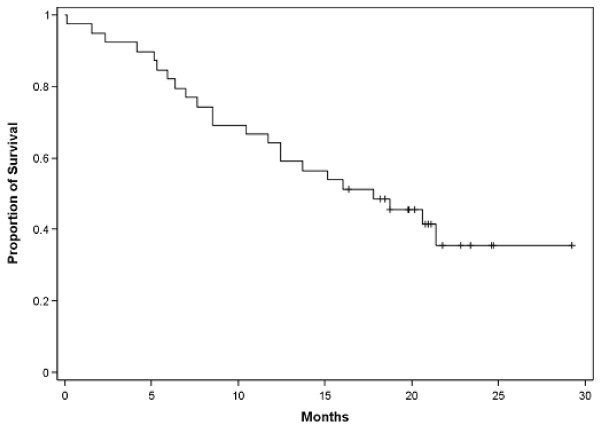
**Survival in patents with platinum refractory or resistant epithelial ovarian cancer**.

Twenty patients (51.3%) had SD resulting in a disease stabilization rate (DSR) (CR + PRs + SDs) of 76.9% in the ITT population and 80.6% in the EE population. The median duration of SD was 6.4 months. The median PFS was 6.0 months (95% CI, 4.2-7.9) and the median survival was 17.8 months (Table 4; Figures 1 and 2). The percentage of patients alive at 12, 18 and 24 months was 64.1%, 48.6% and 35.5%, respectively.

## Discussion

Patients diagnosed with metastatic ovarian cancer eventually become refractory or resistant to platinum and paclitaxel regimens and are subsequently treated with non-platinum monotherapy. Two approved drugs for the treatment of platinum resistant patients include topotecan and PLD [7]. Combination therapy in platinum refractory or resistant recurrent disease has not been proven to be more effective than single agents and is associated with increased toxicity [26].

In single-agent studies, both canfosfamide and PLD have been shown to be active in patients with platinum and paclitaxel refractory or resistant ovarian cancer. Canfosfamide has shown a response rate of 15.6% in the 3-weekly dose schedule (95% CI, 5.32-32.8) and 19% in the weekly dosing (95% CI, 7-36), and a DSR of 50% in phase 2 studies [20,27]. Pegylated liposomal doxorubicin has been shown to have a response rate of 12.3% (95% CI, 7.2-19%) and a DSR of 40% in the platinum resistant population in a phase 3 randomized study [7,20].

In our phase 2 study, the response rate of 25.6% and DSR of 76.9% supports that the combination regimen is more active in the treatment of platinum resistant ovarian cancer than expected from either agent alone. These results are likely due to the distinct mechanisms of action for each drug, as well as non-overlapping toxicities with prior carboplatin-paclitaxel therapy and canfosfamide's non-cross resistance with platinum and taxanes [18,20,28-31]. Although this is a phase 2 non-randomized study, the encouraging median PFS of 6.0 months (95% CI, 4.2-7.9) and median survival of 17.8 months, compare favorably with single agent phase 3 data reported for PLD of 2.1 months and 8.2 months, or for topotecan of 3.1 months and 9.5 months, respectively [7]. The improvement in all efficacy parameters was comparable for patients who had platinum refractory or primary platinum resistant disease of the poorest prognosis and for patients who had secondary platinum resistant disease.

This phase 2 trial is the first to characterize the safety and efficacy of canfosfamide in combination with PLD. The toxicity of PLD is distinct with the most common AEs related to PPE, stomatitis, and hematologic toxicity (primarily neutropenia) [7]. The most common AEs for single agent canfosfamide are: grade 1-2 nausea and vomiting well controlled with standard antiemetics, transient fatigue, and generally no clinically-significant myelosuppression at the recommended dose and dose schedule [20].

In this study, the most common non-hematologic AEs related to PLD were stomatitis (53.8%) and PPE (48.7%). The grade 3-4 hematologic AEs related to the PLD plus canfosfamide combination therapy were: neutropenia (59.0%), leucopenia (43.6%), thrombocytopenia (25.6%), anemia (15.4%), and febrile neutropenia (5.1%). Hematologic AEs were well managed with dose reductions and growth factor support. There were no reports of treatment-related sepsis. Non-hematologic AEs were mild to moderate nausea and vomiting. Other non-hematologic AEs were of a similar grade and frequency as expected for each agent alone. No unexpected hematologic, non-hematologic, or cumulative toxicities were reported.

Several phase 3 studies using canfosfamide have been completed and reported. A randomized phase 3 study (ASSIST-1) of canfosfamide single agent versus PLD or topotecan as third-line therapy in patients with platinum resistant ovarian cancer did not meet the primary survival endpoint [32]. Overall survival was significantly higher in the control arm than in the investigational arm. In a subgroup analysis, PFS and overall survival were also higher with PLD than with topotecan. It was hypothesized that the heterogeneity of cancer biology in third-line therapy patients may have led to variations in the activation or metabolism of canfosfamide, and subsequent anti-cancer therapies may have confounded the survival analysis.

A randomized phase 3 trial (ASSIST-3) with canfosfamide in combination with carboplatin versus PLD as second-line therapy in platinum resistant OC was presented [33]. In this study, the primary endpoint was ORR and the secondary endpoint was PFS. By central blinded IRR, 25% of patients discontinued treatment without documented tumor progression due to difficulties in reading CT/MRI images in ovarian cancer and applying RECIST in this recurrent disease setting. Overall ORR varied between the clinician and IRR assessments, making the ORR indeterminate. Overall median PFS was 3.5 months for both the combination treatment of canfosfamide plus carboplatin and the control arm of PLD alone. In an exploratory analysis, the drug-free period (DFP) ≥ 6 months was identified as a significant prognostic factor for PFS. In this subgroup, 38 patients (19 on the canfosfamide plus carboplatin arm and 19 on the PLD arm) had a DFP of ≥ 6 months. The groups were similar in all demographics and key ovarian cancer disease characteristics. The median PFS in the DFP ≥ 6 months group for canfosfamide plus carboplatin was 7.1 months as compared to 3.5 months on the PLD arm (HR 0.58, p = 0.11). Median survival in the subgroup was 23.4 months on the canfosfamide plus carboplatin arm as compared to 12.9 months on the PLD arm (HR 0.37, p = 0.01). Studies are ongoing in platinum resistant human ovarian cancer cells to analyze changing patterns of genetic expression following exposure to platinum and to understand the optimal DFP following platinum exposure and its relationship to best synergistic response following canfosfamide and carboplatin.

Results of a randomized phase 3 study (ASSIST-5) of canfosfamide in combination with PLD versus PLD as second-line therapy in platinum resistant OC patients was reported [31] [Vergote, I, Finkler, N, Hall, J, et al. Randomized Phase III Study of Canfosfamide in Combination with Pegylated Liposomal Doxorubicin (PLD) as Compared to PLD Alone in Platinum Resistant Ovarian Cancer: Submitted]. This multinational study had randomized 125 patients when the study was temporarily placed on clinical hold to review the results of the above aforementioned trial single-agent canfosfamide trial. The study was allowed to resume enrollment, however, the sponsor decided not to enroll additional patients. The original study was planned for 244 patients. The interim analysis became the final analysis. The median PFS was 5.6 months for canfosfamide plus PLD (n = 65) versus 3.7 months for PLD (n = 60) [HR 0.92, p = 0.7243]. A pre-planned subgroup analysis showed that 75 patients with platinum refractory or primary platinum resistant ovarian cancer had a median PFS of 5.6 months for canfosfamide plus PLD versus 2.9 months for PLD (HR 0.55, p = 0.0425). Hematologic adverse events were 66% on the canfosfamide plus PLD arm versus 44% on the PLD arm, manageable with dose reductions. Non-hematologic adverse events were similar for both arms. The incidence of PPE and stomatitis was lower on the canfosfamide plus PLD arm (23%, 31%, respectively) versus (39%, 49%, respectively) on the PLD arm. The overall median PFS showed a positive trend but was not statistically significant. The median PFS in the platinum refractory and primary platinum resistant patients was significantly longer for canfosfamide plus PLD versus PLD. Canfosfamide may ameliorate the PPE and stomatitis known to be associated with PLD.

In summary, the phase 3 results are consistent with the canfosfamide plus PLD regimen phase 2 results presented in this paper. Further study is planned with canfosfamide in combination with PLD, an active, well tolerated regimen in patients with platinum refractory and primary platinum resistant ovarian cancer.

## Competing interests

JJK is a consultant for Telik, Inc. RCB is a consultant for Telik, Inc. and Fujirebio Diagnostics, Inc. JJK, CFL, PTR, JLW, and CLM declare that they have no competing interests.

MRJ, LM and GLB are employed by Telik, Inc.

## Authors' contributions

MRJ, LM and GLB designed the research protocol. JJK, CFL, PTR, JLW, CLM, RCB were involved in treating patients and collecting data; LM conducted the statistical analysis; MRJ, LM and GLB wrote the paper with contributions from the other authors. All authors read and approved the final manuscript.

## References

[B1] Jemal A Siegel R Ward E Hao Y Xu J Thun MJ Cancer Statistics, 2009 CA: A Cancer Journal for Clinicians 2009 59 4 225 49 10.3322/caac.20006 19474385

[B2] Bristow RE Tomacruz RS Armstrong DK Trimble EL Montz FJ Survival effect of maximal cytoreductive surgery for advanced ovarian carcinoma during the platinum era: a meta-analysis J Clin Oncol 2002 20 5 1248 59 10.1200/JCO.20.5.1248 11870167

[B3] Du Bois A Quinn M Thigpen T 2004 consensus statements on the management of ovarian cancer: Final document of the 3rd International Gynecologic Cancer Intergroup Ovarian Cancer Consensus Conference (GCIG OCCC 2004) Ann Oncol 2005 16 Suppl 8 viii7 viii12 10.1093/annonc/mdi961 16239238

[B4] Agarwal R Kaye SB Ovarian cancer: Strategies for overcoming resistance to chemotherapy Nat Rev Cancer 2003 3 7 502 16 10.1038/nrc1123 12835670

[B5] Thigpen T Design Issues in Clinical Trials of Ovarian Carcinoma FDA and the American Society of Clinical Oncology (ASCO), with co-sponsorship by the American Association for Cancer Research (AACR), public workshop on endpoints for ovarian cancer 2006 http://www.fda.gov/downloads/AboutFDA/CentersOffices/CDER/ucm120669.pdf

[B6] Gordon AN Tonda M Sun S Rackoff W Long-term survival advantage for women treated with pegylated liposomal doxorubicin compared with topotecan in a phase 3 randomized study of recurrent and refractory epithelial ovarian cancer Gynecol Oncol 2004 95 1 1 8 10.1016/j.ygyno.2004.07.011 15385103

[B7] Gordon AN Fleagle JT Guthrie D Parkin DE Gore ME Lacave AJ Recurrent epithelial ovarian carcinoma: A randomized phase III study of pegylated liposomal doxorubicin versus topotecan J Clin Oncol 2001 19 14 3312 22 11454878 10.1200/JCO.2001.19.14.3312

[B8] Lyttle MH Satyam A Hocker MD Glutathione-*S*-transferase activates novel alkylating agents J Med Chem 1994 37 10 1501 7 10.1021/jm00036a016 8182709

[B9] Satyam A Hocker MD Kane-Maguire KA Morgan AS Villar HO Lyttle MH Design, synthesis, and evaluation of latent alkylating agents activated by glutathione *S*-transferase J Med Chem 1996 39 8 1736 47 10.1021/jm950005k 8648613

[B10] Gate L Tew KD Glutathione S-transferases as emerging therapeutic targets Expert Opin Ther Targets 2001 5 4 477 89 10.1517/14728222.5.4.477 12540261

[B11] Montali JA Wheatley JB Schmidt DE Jr Comparison of glutathione S-transferase levels in predicting the efficacy of a novel alkylating agent Cell Pharmacol 1995 2 241 7

[B12] Moscow JA Fairchild CR Madden MJ Expression of anionic glutathione- *S*-transferase and P-glycoprotein genes in human tissues and tumors Cancer Res 1989 49 6 1422 8 2466554

[B13] Tew KD Monks A Barone L Glutathione-associated enzymes in the human cell lines of the National Cancer Institute Drug Screening Program Mol Pharmacol 1996 50 1 149 59 8700107

[B14] Meng F Brown GL Keck JG TLK286-induced activation of JNK-dependent apoptotic signaling pathway Proceedings of the AACR-NCI-EORTC International Conference on Molecular Targets and Cancer Therapeutics; October 29-November 2, 2001; Miami Beach 67 Abstract #326

[B15] Meng F Kim S Brown GL TLK286-induced activation of the stress response apoptotic signaling pathway Proceedings of the Annual Meeting of the American Association for Cancer Research; April 6-10, 2002; San Francisco, California 963 Abstract #4772

[B16] Morgan AS Sanderson PE Borch RF Tumor efficacy and bone marrow-sparing properties of TER286, a cytotoxin activated by glutathione *S*-transferase Cancer Res 1998 58 12 2568 75 9635580

[B17] Rosario LA O'Brien ML Henderson CJ Wolf CR Tew KD Cellular response to a glutathione *S*-transferase P1-1 activated prodrug Mol Pharmacol 2000 58 1 167 74 10860939 10.1124/mol.58.1.167

[B18] Townsend DM Shen H Staros AL Gate L Tew KD Efficacy of a glutathione *S*-transferase p-activated prodrug in platinum-resistant ovarian cancer cells Mol Cancer Ther 2002 1 12 1089 95 12481432 PMC6522260

[B19] Xu H Namgoong S-Y Roth E Synergistic effect of TELCYTA™ (TLK286) in combination with paclitaxel, doxorubicin, carboplatin, oxaliplatin, cisplatin, docetaxel, gemcitabine and iressa in human cancer cells Proceedings of the American Association for Cancer Research; July 11-14, 2003; Washington DC 462 Abstract 2008

[B20] Kavanagh JJ Gershenson DM Choi H Multi-institutional phase 2 study of TLK286 (TELCYTA™, a glutathione *S*-transferase P1-1 activated glutathione analog prodrug) in patients with platinum and paclitaxel refractory or resistant ovarian cancer Int J Gynecol Cancer 2005 15 4 593 600 10.1111/j.1525-1438.2005.00114.x 16014111

[B21] Therasse P Arbuck SG Eisenhauer EA New guidelines to evaluate the response to treatment in solid tumors J Natl Cancer Inst 2000 92 3 205 16 10.1093/jnci/92.3.205 10655437

[B22] Ban N Takahashi Y Takayama T Transfection of glutathione *S*-transferase (GST)-p antisense complementary DNA increases the sensitivity of a colon cancer cell line to adriamycin, cisplatin, melphalan, and etoposide Cancer Res 1996 56 15 3577 82 8758929

[B23] Kavanagh J Kudelka A Lewis L Phase 1-2a study of TLK286 (Telcyta™, a novel glutathione analog) in combination with carboplatin in platinum refractory or resistant (>/= 3rd line) ovarian cancer Proceedings of the AACR-NCI-EORTC International Conference on Molecular Targets and Cancer Therapeutics; November 17-21, 2003; Boston, MA 222 Abstract C155

[B24] NCI National Cancer Institute Common Toxicity Criteria (CTC) 1999 DCTD, NCI, NIH, DHHS

[B25] Kaplan EL Meier P Nonparametric estimation from incomplete observations J Am Stat Assoc 1958 53 282 457 81 10.2307/2281868

[B26] Ozols RF Rubin SC Thomas G Robboy S Hoskins WJ, Perez CA, Young RC Epithelial Ovarian Cancer Principles and Practice of Gynecologic Oncology 1997 2 Philadelphia: Lippincott-Raven Publishers 919 86

[B27] Kavanagh J Kudelka A Spriggs D Phase 2 study of TLK286 (GST P1-1 activated glutathione analog) in patients with platinum and paclitaxel refractory/resistant advanced epithelial ovarian cancer Proceedings of the Biennial Meeting of the International Gynecologic Cancer Society; October 20-24, 2002; Seoul, Korea 530 Abstract OV034

[B28] Izbicka E Lawrence R Cerna C Von Hoff DD Sanderson PE Activity of TER286 against human tumor colony-forming units Anticancer Drugs 1997 8 4 345 8 10.1097/00001813-199704000-00006 9180387

[B29] Wang Z Keck JG Brown GL TLK286, a novel glutathione analog prodrug, induces resensitization to carboplatin in human ovarian cancer, OVCAR3, platinum resistant cells Proceedings of the American Association for Cancer Research; April 16-20, 2005; Anaheim, CA 351 2 Abstract 1500

[B30] Xu H Grossman E Namgoong S-Y Enhanced antitumor activity of TLK286 in combination with oxaliplatin, carboplatin, doxorubicin, paclitaxel and docetaxel in human colorectal, ovarian and breast cancer cell lines Proceedings of the American Association for Cancer Research; April 5-9, 2003; Toronto, Canada 390 1 Abstract 1722

[B31] Vergote I Finkler N Hall J Randomized Phase 3 Study of Canfosfamide (C, TLK286) plus Pegylated Liposomal Doxorubicin (PLD) vs PLD as Second-line Therapy in Platinum (P) Refractory or Resistant Ovarian Cancer (OC) Proceedings of the American Society of Clinical Oncology; May 29-June 2, 2009; Orlando, FL289s Abstract 5552

[B32] Vergote I Finkler N del Campo J Phase 3 Randomized Study of Canfosfamide (Telcyta, TLK286) versus Pegylated Liposomal Doxorubicin or Topotecan as Third-Line Therapy in Patients with Platinum Refractory or Resistant Ovarian Cancer Eur J Cancer 2009 45 2324 32 10.1016/j.ejca.2009.05.016 19515553

[B33] Rose P Edwards R Finkler N Phase 3 study: Canfosfamide (C, TLK286) plus carboplatin (P) vs liposomal doxorubicin (D) as 2nd line therapy of platinum (P) resistant ovarian cancer (OC) Proceedings of the Annual Meeting of the American Society of Clinical Oncology; June 1-5, 2007; Chicago, IL281s Abstract #LBA5529

